# Hybrid Additive Manufacturing of Fused Filament Fabrication and Ultrasonic Consolidation

**DOI:** 10.3390/polym14122385

**Published:** 2022-06-12

**Authors:** Wenzheng Wu, Haiming Wang, Jiaqi Wang, Qingping Liu, Zheng Zhang, Ke Li, Yuhan Gong, Ji Zhao, Luquan Ren, Guiwei Li

**Affiliations:** 1 Advanced Materials Additive Manufacturing ((AM)2) Lab, School of Mechanical and Aerospace Engineering, Jilin University, Changchun 130025, China; wzwu@jlu.edu.cn (W.W.); wanghm19@mails.jlu.edu.cn (H.W.); wjq19@mails.jlu.edu.cn (J.W.); zhangzheng20@mails.jlu.edu.cn (Z.Z.); like20@mails.jlu.edu.cn (K.L.); gongyh9919@jlu.edu.cn (Y.G.); lqren@jlu.edu.cn (L.R.); 2 Chongqing Research Institute, Jilin University, 618 Liangjiang Avenue, Longxing Town, Yubei District, Chongqing 401122, China; 3 Key Laboratory of Bionic Engineering (Ministry of Education), Jilin University, Changchun 130022, China; liuqp@jlu.edu.cn; 4 School of Mechanical Engineering and Automation, Northeastern University, Shenyang 110004, China; jzhao@jlu.edu.cn

**Keywords:** fused filament fabrication, ultrasound strengthening, mechanical properties, heterogeneous material, hybrid additive manufacturing, 3D printing

## Abstract

Fused filament fabrication (FFF) additive manufacturing technology has the advantages of being low cost, having a simple operation, using wide types of molding materials, and producing less pollution during the printing process. However, the mechanical properties of the molded sample are unsatisfactory due to the limited bonding force between the filaments during the forming process, which limits its further development and application in the engineering field. Herein, the hybrid additive manufacturing technology for heterogeneous materials based on the ultrasonic-assisted enhanced fused filament fabrication technology was proposed. The mechanism of ultrasonic vibration on the strengthening of FFF samples was explored. The influence mechanisms of bonding time and ultrasonic strengthening times, ultrasonic strengthening and static load compression on the strengthening of mechanical properties of the sample were investigated. The effects of the thickness and printing angle of the FFF samples on the ultrasonic-enhanced mechanical properties were explored. The tensile strength of the one-time ultrasonic-strengthened sample is up to 43.43 MPa, which is 16.12% higher than that of the original. The maximum bending strength of the four-time ultrasonic-strengthened sample is 73.38 MPa, which is 78.98% higher than that of the original. Ultrasonic strengthening not only re-fused the pores inside the sample, but also improved the bond strength between the rasters. With the increase in the thickness of the sample, the increase rate of ultrasonic to the strength of the sample decreased significantly. The effects of ultrasound on the interlayer adhesion of samples with various printing angles were different. Based on the systematic research on the influence mechanism of ultrasonic process parameters and molding process parameters on the strengthening of FFF, a molding method for additively manufacturing heterogeneous material parts while strengthening the mechanical properties of FFF samples was proposed, and the influence mechanisms of the molding process on the mechanical properties and shape memory properties of the sample were explored, which can broaden the application of FFF technology in the engineering field.

## 1. Introduction

Fused filament fabrication (FFF), as an early and widely used additive manufacturing technology, which has the advantages of having a simple operation, being low-cost and low-pollution, is widely used in aerospace, automobile, biomedical, daily life and other fields [1,2,3,4,5,6,7,8,9]. However, due to the process characteristics of raster-by-raster and layer-by-layer cumulative bonding in the FFF process, there are defects such as pores between rasters and weak bonding between rasters, resulting in low mechanical properties of the molded samples [10,11,12,13]. Improving the mechanical properties of molded samples has become the focus of research in the field of FFF.

Ning et al. prepared carbon fiber reinforced composites by adding different contents of carbon fibers to ABS for fused deposition printing, and conducted experimental studies on the mechanical properties of the molded samples [14]. Compared with the pure material samples, the addition of carbon fibers can increase the tensile strength and Young’s modulus of the specimens, but may reduce toughness, yield strength, and ductility. Weng et al. prepared ABS/OMMT (organically modified montmorillonite) nanocomposites, and conducted experimental research on fused deposition printing, and found that the addition of OMMT can significantly improve the mechanical properties of fused deposition ABS samples, 5wt% OMMT increases its tensile strength by 43%, and the introduction of OMMT into ABS can reduce the linear expansion rate of the material and reduce the warpage deformation of the sample during the fused deposition molding process [15]. Jiang et al. used the high-performance polymer polyetherimide (PEI) as the material for FFF 3D printing, which has a high glass transition temperature and good mechanical properties [16]. The experiments showed that the optimal nozzle temperature is at 370 °C, and the tensile strength of the sample is 104 MPa, which is only 7% lower than that of the injection molded part. Chaco’n et al. studied the effects of build direction, layer thickness and feed rate on the mechanical properties of fused-deposited PLA, and found that the strength and stiffness of the samples in the vertical direction were lower, and the mechanical properties increased with the increase in layer thickness and progress increased as the rate decreased [17]. Durgun et al. used ABS as the printing material to study the effect of the construction direction and forming angle of the part on the mechanical properties of the FFF samples [18]. Osman–developed ABS-RS (straw) composites for FFF process and tested the effect of RS content on mechanical properties [19].

The above studies are listed in Table 1. The above studies on improving the mechanical properties of FFF samples mainly focus on the optimization of printing parameters and the modification of printing materials. The use of an ultrasonic plastic welding machine to apply ultrasonic vibration to the FFF samples for post-processing can quickly improve the mechanical properties of the samples without changing the raw materials [20,21,22]. It has the advantages of taking up a short amount of time, having a simple operation and producing no pollution [23,24,25].

The basic principle of ultrasonic plastic welding is that when the ultrasonic wave acts on the plastic surface, the upper and lower plastic samples rub against each other and generate local high temperature at the contact surface. Due to the poor thermal conductivity of the plastic, the heat cannot be dissipated in time, so the contact surface melts rapidly. Welds are formed under the action. Due to the advantages of no pollution and no need for any adhesive, ultrasonic plastic welding technology is widely used in automotive, aerospace, medical, electronics and other fields. Choudhury et al. carried out ultrasonic welding of green composite materials with bamboo fiber as reinforcement and polylactic acid as a matrix material, and explored the effects of welding time, holding time and welding pressure on the tensile failure load of welded specimens [26]. Tao et al. explored the effect of ultrasonic welding time on the welding strength of carbon fiber reinforced PEEK composites. The study showed that with the increase in welding time, the welding strength first increased and then decreased, and 0.9 s was the optimal welding time [27]. Wang et al. used short carbon fiber-reinforced PA6 composites as ultrasonic welding materials to study the effect of different energy inputs on the welding performance [28]. The above studies illustrate that ultrasonic welding is a method used to melt the gaps among the samples, which can be applied to a polymer or its composites. Therefore, ultrasonic vibrations can be applied to strengthen the mechanical properties of a polymer and its composite parts.

In this paper, polylactic acid (PLA) was used as the printing material, and the mechanism of ultrasonic enhancement of the mechanical properties of the FFF samples was studied in detail. The welding effect of ultrasonic was used to carry out a step-by-step study on the FFF two-dimensional samples. Layer welding and strengthening, and embedding the metal to realize the additive manufacturing of metal and polymer composites were carried out, which is of great significance in terms of broadening the application prospects of FFF [29].

## 2. Experimental and Methods

### 2.1. Mechanism of Mechanical Properties of Ultrasonic-Enhanced FFF Samples

#### 2.1.1. Experimental Design

Using an ultrasonic plastic welding machine to apply ultrasonic energy to the samples after FFF can improve their mechanical properties, and can also improve experimental study on the mechanism of the action of ultrasound. The single factor experiment method was used, and the experimental scheme is shown in Table 2.The first group of experiments took the number of ultrasonic strengthening as the variable, the sample thickness was 3 mm, and the sample printing angle was 0°; the second group of experiments took the sample thickness as the variable, the sample printing angle was 0°, and ultrasonic was applied to the sample once; the third group of experiments used the printing angle as a variable, the thickness of the sample was 3 mm, and ultrasonic was applied to the sample once.

By repeatedly applying ultrasonic vibration to the sample, the inside of the sample undergoes a cycle process of heating, cooling, and heating again, which will affect the fusion of the internal wires, thereby affecting the mechanical properties of the sample; during the ultrasonic strengthening process, the sample is placed horizontally in the bottom mold, and the ultrasonic system transmits ultrasonic energy to the inside of the sample through the welding head. The ultrasonic transmission direction is the vertical direction, and it is transmitted along the thickness direction of the sample. The ultrasonic vibrations are transmitted from the contact surface between the sample and the welding head to the contact surface with the bottom mold. The thickness of the sample will affect the penetration ability of ultrasonic waves inside the sample, which will further affect the improvement effect of ultrasonic strengthening on the mechanical properties of the sample. The printing angle refers to the angle between the sample and the substrate during the molding process. Due to the printing layers, bonding directions of the samples differ depending on the printing angles, and the fusion effect of ultrasonic on the inner wire and the bonding effect between layers will be different, resulting in varying effects of ultrasonic on improving the mechanical properties of the samples with different printing angles. It is of great significance to explore the mechanism of ultrasonication by studying the effects of ultrasonic strengthening times, sample thickness and printing angle on the mechanical properties of ultrasonically strengthened samples. After the sample was ultrasonically strengthened, the thickness decreased due to the fusion of the wires. At room temperature, the electronic universal testing machine was used to continuously press the sample to the same thickness as the ultrasonic strengthening sample. The tensile strength of the statically loaded compressive sample and the ultrasonically strengthened sample was compared.

#### 2.1.2. Experiment Procedure

(1) FFF Sample Preparation

In the experiment, the EinStart-S desktop FFF printer (SHINING 3D Co., Ltd., Hangzhou, China) was used for printing. The 3D modeling software was applied to design the 3D model of the sample, and the model was imported into the slicing software 3Dstart to generate a gsd file for printing.

**Figure 1:**
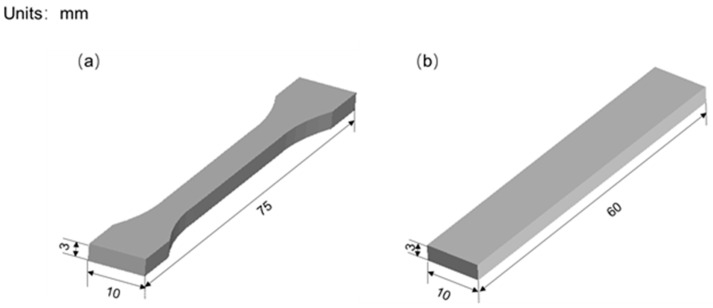
Mechanical sample model, (**a**) tensile sample, (**b**) bending sample.

Using Solidworks 3D modeling software, the tensile mechanics sample was designed according to GB/T 16421-1996, and the bending mechanics sample was designed according to GB/T 9341-2008. The shape and size of the 3D model of the sample are shown in Figure 1. The selection of printing parameters is shown in Table 3.

(2) Ultrasound enhanced post-processing

**Figure 2:**
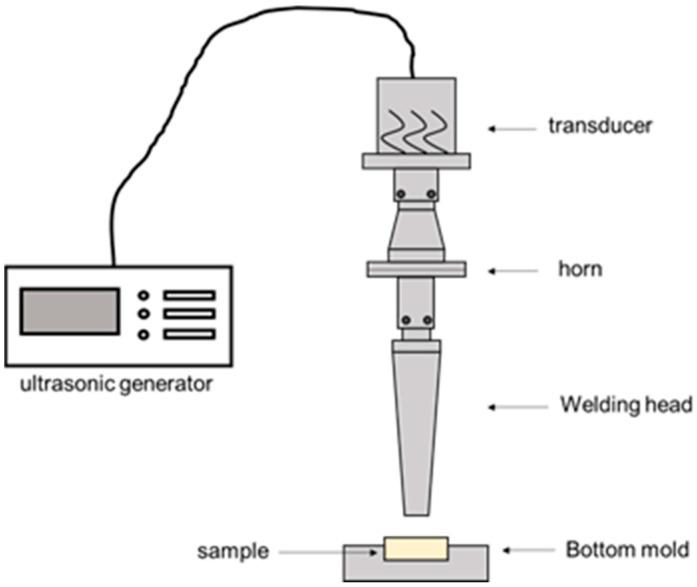
Schematic diagram of ultrasonic strengthening equipment.

As shown in Figure 2, a customized ultrasonic strengthening equipment was used for experiments. It is mainly composed of ultrasonic generator, transducer, horn, welding head and bottom mold. The ultrasonic generator converts the electric energy of 50–60 Hz into high-frequency electric energy of 20–40 KHz, and then transmits it to the ultrasonic transducer. The most widely used frequency for ultrasonic plastic welding is 20 KHz. The function of the transducer is to convert the high-frequency electric energy from the ultrasonic generator into mechanical vibration of the same frequency. The amplitude of the mechanical vibration transmitted by the transducer is transmitted by the horn. Under a certain pressure, the aluminum alloy welding head injects ultrasonic energy into the plastic sample. There are defects such as pores or weak bonding between the wires inside the sample. The high-frequency ultrasonic vibration causes friction, deformation and limited temperature rise, and fusion forming defects. The aluminum alloy bottom mold plays a role in fixing and supporting the sample to prevent the sample from shifting during the ultrasonic vibration process.

**Figure 3:**
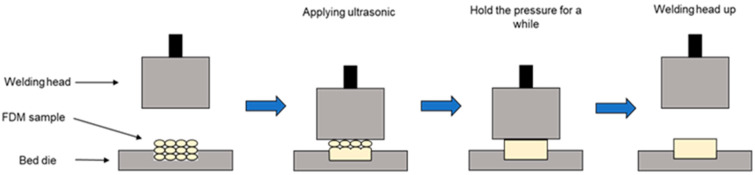
Schematic diagram of ultrasonic strengthening process.

The ultrasonic strengthening process of the sample is shown in Figure 3. First, the sample is fixed on the bottom mold, the appropriate ultrasonic strengthening process parameters are set, the ultrasonic strengthening system is started, the welding head is lowered to contact the sample, and then ultrasonic vibration and a certain pressure are applied to the sample. After ultrasonic strengthening, the welding head holds pressure on the sample for a period of time to prevent warping of the sample. Finally, the welding head rises, and the entire ultrasonic strengthening process ends.

The selection of ultrasonic process parameters is shown in Table 4. On this basis, the effects of one-time ultrasonic welding with a welding time of 0.5 s and a second ultrasonic welding time of 0.25 s on the mechanical properties of the samples were compared.

(3) Mechanical property test

The mechanical properties of the original and ultrasonically strengthened samples were tested by a universal testing machine. The tensile test speed was 1 mm/min, the bending test speed was 2 mm/min, and the bending test span was 48 mm.

### 2.2. Experimental of Hybrid Additive Manufacturing Based on Ultrasonic Welding

The printing parameters of the experimental samples in this section are consistent with Table 3, and the ultrasonic process parameters are consistent with Table 4.

#### 2.2.1. Research on Mechanical Properties

(1) Ultrasonic welding of FFF samples

**Figure 4:**
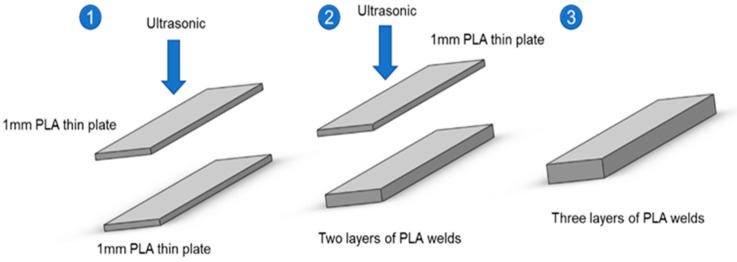
Schematic diagram of PLA ultrasonic welding forming process.

A 1 mm × 10 mm × 60 mm sheet was printed with a desktop FFF printer, and ultrasonic welding and strengthening were carried out layer by layer as shown in Figure 4. Two-layer and three-layer PLA welding samples were formed in the experiment, which were recorded as 2 × PLA, 3 × PLA, and the tensile strength and flexural strength of the molded sample were tested.

(2) Metal-embedded ultrasonic welded FFF prototypes

**Figure 5:**
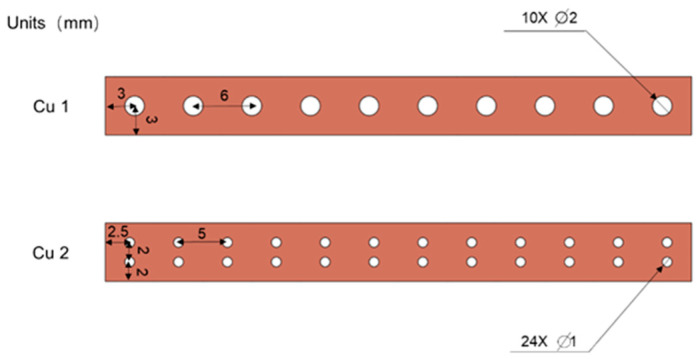
Schematic diagram of the size of the round hole metal strip.

**Figure 6:**
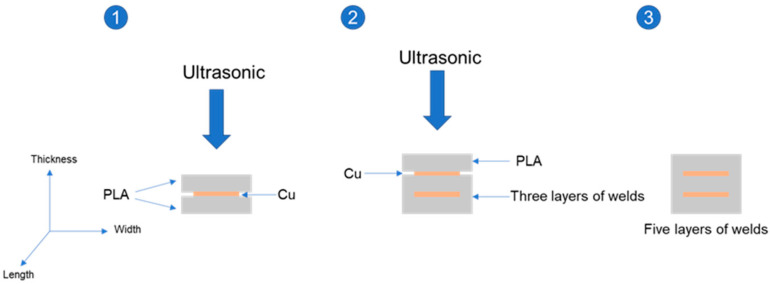
Schematic diagram of the forming process of metal embedded PLA weldment.

A metal copper strip with a size of 0.2 mm × 6 mm × 60 mm was selected, and the laser cutting technology is used to cut circular hole arrays with different diameters and positions on the metal strip, which are, respectively, recorded as Cu1 and Cu2, and their dimensions are shown in Figure 5. In the manner shown in Figure 6, the metal was sandwiched between two layers of PLA with the dimensions of 1 mm × 10 mm × 60 mm, with the metal at the center position, thereby sealing the metal strip in the PLA, forming the metal and PLA mutual for the superimposed samples, and 3-layer and 5-layer samples were formed. According to the number of layers and the embedded metal, the formed samples were abbreviated as 3-layer Cu1, 5-layer Cu1, 3-layer Cu2, and 5-layer Cu2. During the molding process, the PLA can be connected to each other through the round holes, and the metal can be embedded in the polymer. The round holes on the metal strip increase the contact area between the PLAs, so that the upper and lower PLA samples can be welded more firmly. The metal inside achieves a good seal.

#### 2.2.2. Characterization of Shape Memory Performance

**Figure 7:**
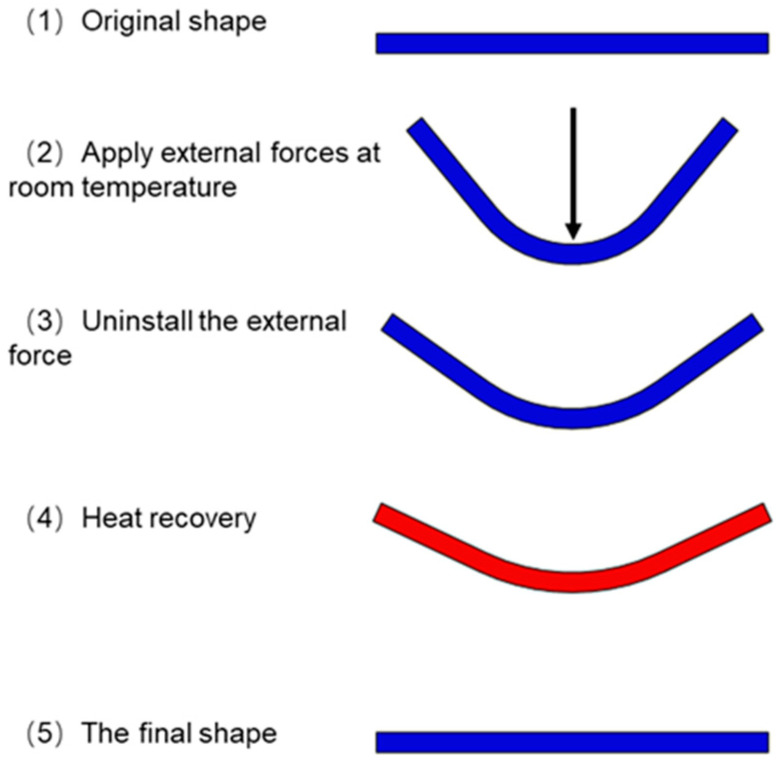
The deformation recovery process of the sample.

The metal-encapsulated PLA weldments were prepared for shape memory experiments. The entire shape memory experiment process is shown in Figure 7: (1) The samples are bent and deformed with a universal testing machine at room temperature. The sample is placed horizontally, the span is 40 mm, the speed of the indenter is 1 mm/min, and the indenter stops moving after bending the sample with a displacement of 15 mm. Pictures are taken to record the bending angle *θ*_0_ of the sample. (2) After keeping the indenter acting on the sample for 10 min, the indenter is removed, the elastic deformation of the sample is restored, and pictures are taken to record the bending angle *θ*_1_ of the sample at this time. (3) The sample is put into a 70 °C hot water bath, the recovery of the sample is observed and recorded with a video recording, and the bending angle *θ*_2_ of the sample is recorded after it has returned to its final shape.

The shape fixation rate *α*_1_, the shape recovery rate *α*_2_ and the shape average recovery rate ν are selected as the characterization parameters, and the calculation formula is as follows:

(1) $$\alpha_{1}=\frac{180-\theta_{1}}{180-\theta_{0}}\times 100\%$$

(2) $$\alpha_{2}=\frac{\theta_{2}-\theta_{1}}{180-\theta_{1}}\times 100\%$$

(3) $$\nu =\frac{\theta_{2}-\theta_{1}}{t}$$

In the formula, *t* is the time it takes for the sample to recover its deformation in a hot water bath, and other symbols have been marked above. In order to increase the accuracy of the experiment, three samples were prepared for each group of experiments, and the average value of the sample shape fixation rate, shape recovery rate, and average shape recovery rate was selected as the final result.

(3) Sample preparation

**Figure 8:**
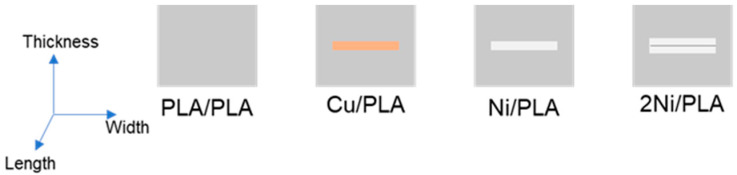
Schematic diagram of the sample used for deformation recovery test.

First, a thin plate of 1 mm × 10 mm × 75 mm was printed with an FFF printer for ultrasonic welding. The welding and forming process of the sample is consistent with the sample preparation process in Section 2.2.1, and the welded part as shown in Figure 8 is prepared: two PLA sheets are simply welded, encapsulating metal copper between the two PLA sheets, the Ni-based amorphous alloy is encapsulated between two PLA sheets, and two pieces of Ni-based amorphous alloy are stacked and encapsulated between two PLA sheets. The above samples are recorded as PLA/PLA, Cu/PLA, Ni/PLA, 2Ni/PLA in turn. The sample materials and dimensions used for welding and forming are shown in Table 5.

## 3. Results and Discussion

### 3.1. Ultrasonic Strengthening Mechanism

#### 3.1.1. Experimental Results

**Figure 9:**
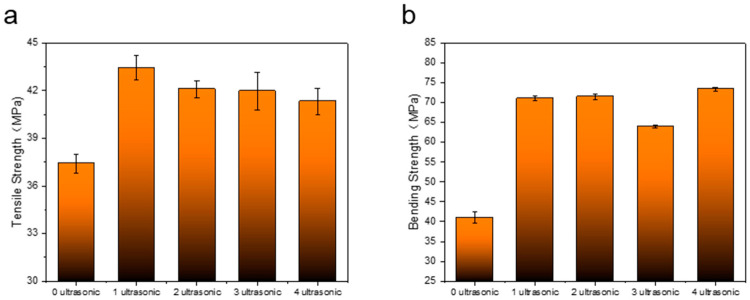
(**a**) Tensile strength of samples with different ultrasonic strengthening times. (**b**) Bending strength of samples with different ultrasonic strengthening times.

The original, primary reinforcement, secondary reinforcement, tertiary reinforcement, and quadruple reinforcement are represented as 0 ultrasonic, 1 ultrasonic, 2 ultrasonic, 3 ultrasonic, and 4 ultrasonic, respectively. As shown in Figure 9a, the tensile strength of the original without ultrasonic strengthening is 37.4 MPa, and the tensile strengths of the samples subjected to 1, 2, 3, and 4 ultrasonic were 43.43 MPa, 42.1 MPa, 41.97 MPa, and 41.32 MPa, respectively. It can be found that with the increase in ultrasonic times, the tensile strength of the sample gradually decreases, but the decrease is not large, and it remains higher than the tensile strength of the original. Compared with the original sample, the tensile strength of the sample with four ultrasonic waves increased at least 10.48%. During an ultrasonic strengthening process, the ultrasonic strengthening system inputs ultrasonic energy into the sample, and the high-frequency mechanical vibration causes friction between the rasters to generate frictional energy. Due to the frictional heating of the raster, the frictional energy is converted into heat energy, and the interior of the sample heats up rapidly, which increases the energy of the molecular chain, thereby enhancing the mobility of the molecular chain and accelerating the diffusion and cross-linking of the molecular chain at the bond of the raster. Under the pressure of the welding head, the raster will re-fuse and become tighter, repairing the defects in the printing process, so that it can better resist tensile deformation and increase the tensile strength. However, if ultrasonic waves are applied to the sample repeatedly, the sample will cycle from heating to cooling and then to heating. During the process of ultrasonic vibration fusing the raster, the original fusion part may also be damaged, so the sample after multiple ultrasonic waves may be damaged. The tensile strength decreased slightly. Due to the fusion of the wire, the overall porosity of the sample decreases, and its size shows that the length and width remain unchanged, and the thickness decreases. The statistics of the thickness reduction rate of the sample are shown in Table 6.

As shown in Figure 9b, the flexural strength of the original without ultrasonic strengthening is 41 MPa and the bending strengths of the ultrasonic samples were 71.05 MPa, 71.5 MPa, 63.96 MPa, and 73.38 MPa, respectively, after applying 1, 2, 3, and 4 times. It is found that the increase in the bending strength of ultrasonic vibration is significantly greater than that of the tensile strength, but there is no obvious change in the bending strength after multiple ultrasonic strengthening. The maximum strength increase is 78.98%. For the three-point bending test, the internal stress of the sample is more complicated than that for the tensile test, because the sample must bear not only tensile stress but also compressive stress during the bending deformation process.

**Figure 10:**
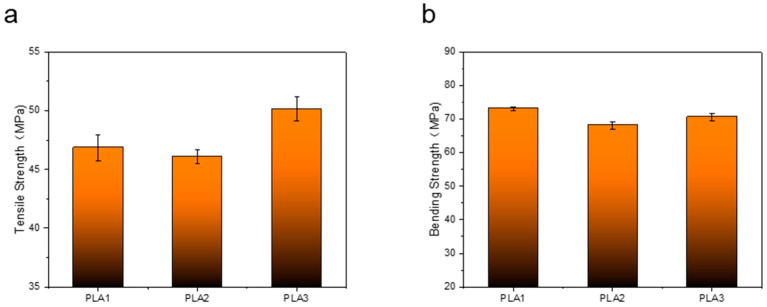
(**a**) Tensile strength of samples under different ultrasonic strengthening methods. (**b**) Bending strength of samples under different ultrasonic strengthening methods.

The samples whose welding time is 0.25 s for primary strengthening, 0.25 s for secondary strengthening, and 0.5 s for primary strengthening are abbreviated as PLA1, PLA2, and PLA3, respectively. As shown in Figure 10a, the tensile strengths of the first ultrasonically strengthened sample with a welding time of 0.25 s, 0.25 s of the second ultrasonically strengthened sample, and 0.5 s of the first ultrasonically strengthened sample are as follows: 46.87 MPa, 46.08 MPa, 50.15 MPa. The tensile strength of the first ultrasonic sample with a welding time of 0.5 s is higher than that of the second ultrasonic sample with a welding time of 0.25 s. Compared with the first ultrasonic sample with a welding time of 0.25 s, the tensile strength of the second ultrasonic sample with a welding time of 0.25 s decreased by 1.69%, and the tensile strength of the first ultrasonic sample with a welding time of 0.5 s increased by 7%. Although the two strengthening methods applied the same ultrasonic energy to the inside of the sample, the welding time was 0.5 s, and the ultrasonic effect was continuous throughout the whole process. With the continuous input of ultrasonic energy, the rasters were fully fused, and the fusion area continued to expand to resist stretching, meaning that the ability to deform is continuously enhanced; the welding time is 0.25 s for the second ultrasonic wave, and the ultrasonic effect is interrupted. The first ultrasonic wave is applied, and the raster is gradually fused, and the bonding force is enhanced. Applying ultrasound again will damage the previously fused parts, while fusing the raster defects. In addition, the secondary heating of the polymer without sufficient cooling will also adversely affect the properties of the material itself. These are the factors that cause the tensile strength of the second ultrasonic sample with welding time of 0.25 s to be lower than the tensile strength of the first ultrasonic sample with welding time of 0.5 s. As shown in Figure 10b, the welding time is 0.25 s for the first ultrasonic strengthening, 0.25 s for the second ultrasonic strengthening, and 0.5 s for the first ultrasonic strengthening. The bending strength of the samples are 73.08 MPa, 68.11 MPa, 70.6 MPa, respectively. Although the flexural strength of the 0.25 s secondary ultrasonic and 0.5 s primary ultrasonic samples decreased compared with the 0.25 s primary ultrasonic sample, the flexural strength of the 0.5 s primary ultrasonic sample was higher than that of the 0.25 s primary ultrasonic sample. It shows that under the condition of the same input ultrasonic energy, the continuous ultrasonic effect is stronger than the discontinuous ultrasonic effect on the ability of the sample to resist bending deformation.

**Figure 11:**
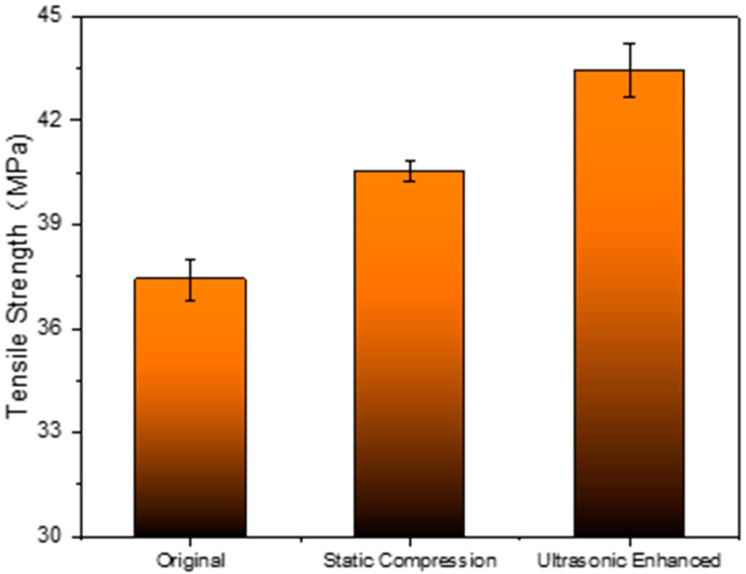
Comparison of static load compression and ultrasonic strengthening tensile strength.

Under static load compression, the tensile strength of the sample will also be improved to a certain extent, but the increase is significantly smaller than that of ultrasonic strengthening. As shown in Figure 11, the tensile strength of the sample after static load compression is 40.54 MPa, which is 8.4% higher than the original tensile strength of 37.4 MPa. After ultrasonic strengthening, the tensile strength is 43.43 MPa, which is 16.12% higher than that of the original, which is almost twice the increase rate of static load compressive strength.

**Figure 12:**
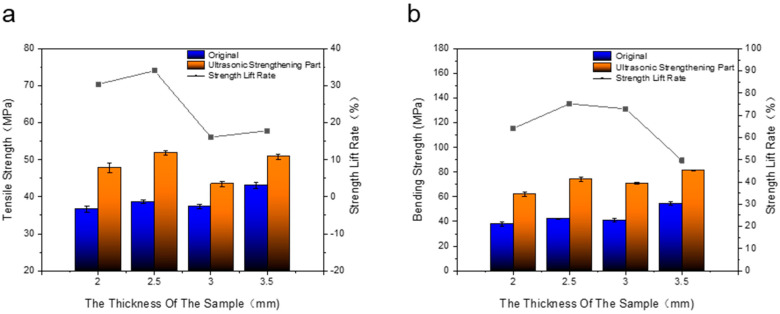
(**a**) Tensile strength of samples with different thicknesses before and after ultrasonic strengthening. (**b**) Bending strength of samples with different thicknesses before and after ultrasonic strengthening.

As shown in Figure 12a, after the samples with thickness of 2 mm, 2.5 mm, 3 mm and 3.5 mm were subjected to ultrasonic action in the same way and parameters, the tensile strength improvement rates were 30.42%, 34.06%, 16.12%, and 17.84%, respectively. It can be seen that when the thickness of the sample is 3 mm and 2.5 mm, the tensile strength increase rate is significantly lower than that of the 2 mm and 2.5 mm thick samples. During the printing process of the sample, it is printed layer by layer along the thickness direction of the sample, and the layers are combined by the bonding force of the raster material. Due to the obstruction of the raster, the energy will be gradually weakened. When the thickness of the sample is large, the ultrasonic energy will be reduced to a greater degree, and the ultrasonic wave will have a small effect on the improvement of the adhesion between the layers. The increase in tensile strength is lower than that of samples with smaller thickness. As shown in Figure 12b, for the original without ultrasonic strengthening, the maximum bending strength of the sample with a thickness of 3.5 mm is 54.27 MPa. After ultrasonic strengthening post-treatment in the same way and with the same parameters, the increase rates of bending strength of samples with thickness of 2 mm, 2.5 mm, 3 mm and 3.5 mm are 64.14%, 75.23%, 72.9% and 49.7%, respectively. With the increase in the thickness of the sample, it first increased and then decreased, and the increase rate of the bending strength of the sample with a thickness of 3.5 mm by ultrasonic strengthening was the lowest. The bending strength is mainly related to the interlayer bonding force of the sample. After the sample is ultrasonically strengthened, multiple printing layers are fused into a deposition layer, the maximum bending load that can be endured per unit area increases, and the bending strength will be significantly improved. Within a certain thickness range of the sample, the ultrasonic wave is less hindered during the transmission process inside the sample, and the effect is sufficient. As the thickness of the sample increases, the number of printing layers also increases, and the deposited layer fused by the printing layer resists bending deformation. Therefore, the increase rate of ultrasonic wave on the bending strength of the sample increases; however, the penetration ability of the ultrasonic wave inside the sample is limited. After the thickness of the sample exceeds a certain range, with the increase in the thickness of the sample, the ultrasonic wave is transmitted during the transmission process. The greater the obstacle in the sample, the more the ultrasonic energy will be weakened, and the effect of improving the bonding force between the layers of the sample will become smaller, resulting in a gradual decrease in the increase rate of ultrasonic vibration on the bending strength of the sample.

**Figure 13:**
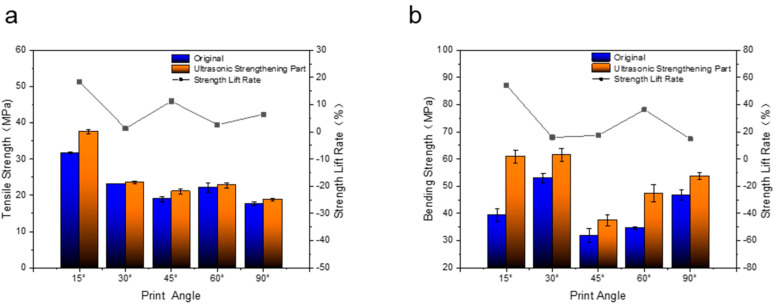
(**a**) Tensile strength of samples with different printing angles before and after ultrasonic strengthening. (**b**) Bending strength of samples with different printing angles before and after ultrasonic strengthening.

As shown in Figure 13a, for the original without ultrasonic strengthening, the tensile strengths of the samples with printing angles of 15°, 30°, 45°, 60°, and 90° are 31.71 MPa, 23.17 MPa, 18.92 MPa, 22.15 MPa, 17.71 MPa, respectively. It was found that the tensile strength of the samples with a printing angle of 45° was lower than that of the samples with a printing angle of 60°, and that the tensile strength of the samples with a printing angle of 15°, 30°, 60° and 90° gradually increased with the increase in the printing angle. The tensile strength of the sample is the lowest when the printing angle is 90°. The reason for this is that the accumulation direction of the printing layers of the samples with different printing angles is inconsistent with the direction of the tensile force. The larger the printing angle, the more it is necessary to rely on the bonding force between the layers to resist the tensile deformation, but the strength of the bond between the layers of the FFF sample is relatively weak, so the tensile strength is low. When the printing angle of the sample is 90°, the direction of the tensile force is consistent with the direction of the bonding force between the layers, so it is easy to break at the bonding between the layers, resulting in the lowest tensile strength. The tensile strength at a printing angle of 45° is lower, probably because the shear stress on the section where the printing layer is located is the largest, and it is easy for the slippage between the printing layers to cause a decrease in tensile strength. For the samples after ultrasonic strengthening, the tensile strength of the samples is consistent with the trend of the original. The tensile strengths of the samples with printing angles of 15°, 30°, 45°, 60°, and 90° are 37.55 MPa, 23.47 MPa, 21.05 MPa, 22.73 MPa, 18.86 MPa, respectively. Compared with the original, the tensile strength improvement rate of the sample with a printing angle of 15° is a maximum of 18.42%; the increase rate of the tensile strength of the sample with a printing angle of 30° and a printing angle of 60° is very small compared with the original, only 1.29% and 2.62%, respectively. There is basically no change; the tensile strength of the sample with a printing angle of 45° and a sample with a printing angle of 90° is increased by 11.26% and 6.49%, respectively, compared with the original. The reason for the difference in the tensile strength improvement rate of the samples with different printing angles is the relationship between the direction of ultrasonic action, the direction of the printed layer, and the direction of the tensile force; as shown in Figure 13b, after ultrasonic strengthening, the bending strength of the samples formed at different printing angles has been relatively significantly improved. The increase rates of ultrasonic on the bending strength of the samples with printing angles of 15°, 30°, 45°, 60°, and 90° were 54.54%, 15.96%, 17.46%, 36.38%, and 14.81%, respectively. The flexural strength improvement rate of the sample with a printing angle of 15° is the largest, and the increase rate of the flexural strength of the sample with a printing angle of 90° is the smallest. However, there is no obvious regularity in the change in the bending strength of the samples with the printing angle before and after ultrasound. The bending strength of the samples with a printing angle of 30° before and after ultrasound is the highest, which are 53.08 MPa and 61.55 MPa, respectively.

#### 3.1.2. Analysis of Tensile Section Topography

**Figure 14:**
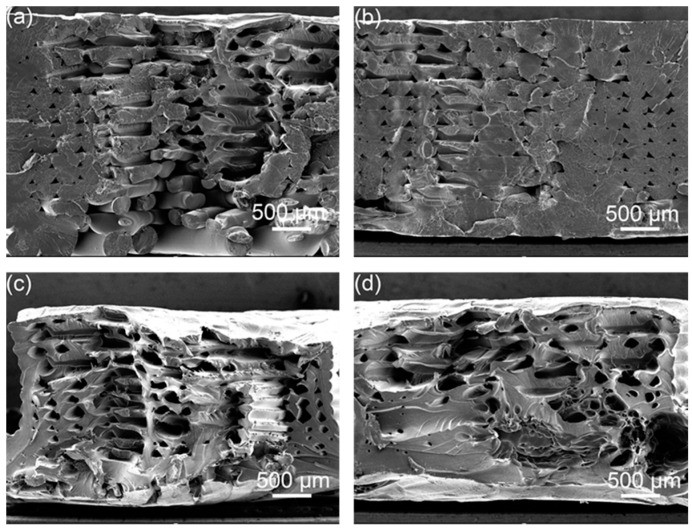
SEM images of the tensile specimen; (**a**) original, (**b**) first ultrasound, (**c**) second ultrasound, (**d**) fourth ultrasound.

It can be seen from the cross-sectional electron microscope of the tensile sample in Figure 14 that the morphology of a single broken raster can be seen in the original without ultrasonic strengthening, indicating that the bonding between the raster and the raster is not strong, and it is subjected to vertical tensile force, cracks are prone to occur in parts with low bond strength and printing defects, resulting in a decrease in tensile strength. As shown in Figure 14b, after one ultrasonic strengthening, the adjacent rasters in some parts are fused, the pores between the rasters are reduced, and they are almost fused together, and the bonding area between the rasters increases, significantly improving the tensile strength. As shown in Figure 14c,d, after multiple ultrasonic strengthening of the sample, the ultrasonic energy is intermittently input into the sample, and it is difficult to distinguish the outline of a single raster. Some parts fuse well, but the pores in some parts become larger under the action of ultrasonic vibration, and these defects will reduce the tensile strength.

**Figure 15:**
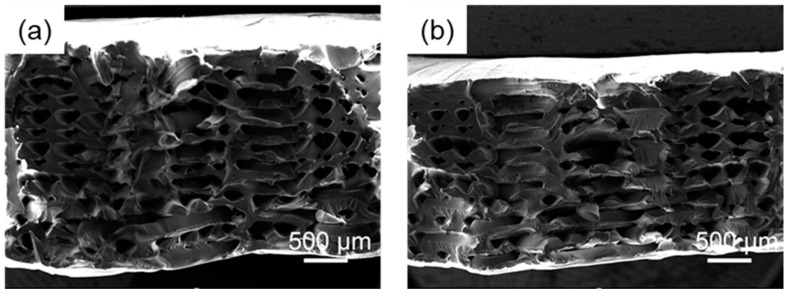
Tensile section, (**a**) 0.25 s second ultrasonic sample, (**b**) 0.5 s first ultrasonic sample.

From the tensile cross-sectional electron microscope image of the sample in Figure 15, it can be found that compared with the 0.25 s second ultrasonic strengthening sample, the 0.5 s primary ultrasonic strengthening sample is more closely bonded between the layers, and some parts are between the upper and lower layers. It has been melted into a whole, and the thickness of the sample is also smaller, which means that in the 0.5 s ultrasonic strengthening process, the ultrasonic effect is continuous, the temperature inside the sample continues to heat up, and the molecular chains at the raster interface can fully diffuse, making the raster fusion more stable.

**Figure 16:**
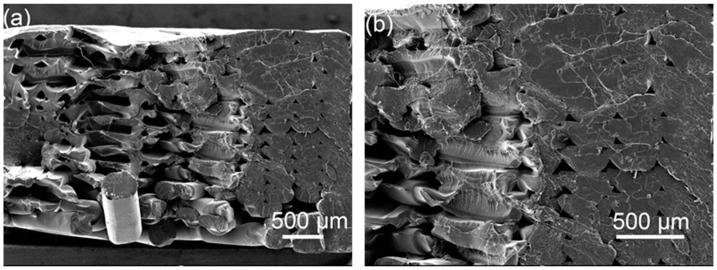
Electron micrograph of the tensile cross-section of the static load compression sample (**a**) 30 times (**b**) 50 times.

It can be seen from Figure 16 that the pores between the rasters are reduced after static load compression, so the tensile strength will be improved to a certain extent, but under static load compression, the internal temperature of the sample will not change, molecular chain mobility will not be enhanced, and the raster will not re-fuse, but, instead, it simply deforms the raster by extrusion and packs it more tightly. The outline of the filament after being stretched and fractured can be clearly seen in the electron microscope image. The bonding force between the filaments at the position with printing defects is still poor, and it is prone to fracture under vertical tension.

**Figure 17:**
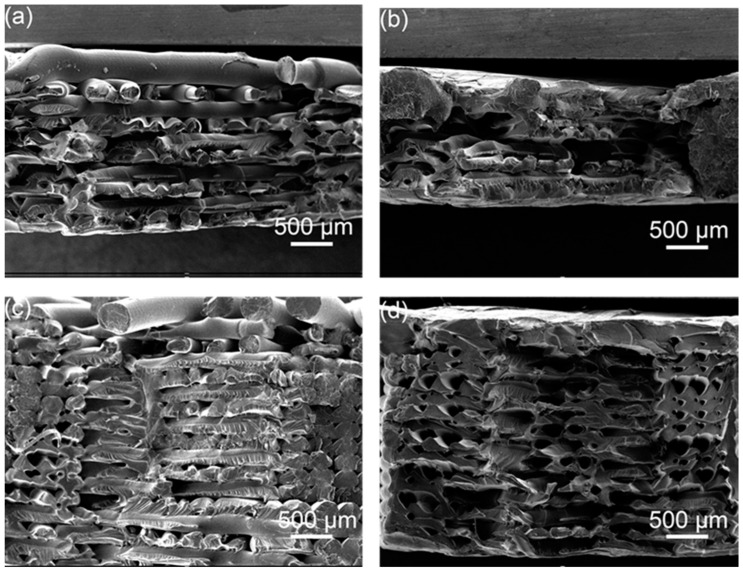
Cross-sectional view of the tensile sample, (**a**) 2 mm original, (**b**) 2 mm ultrasonically strengthened sample, (**c**) 3.5 mm original, (**d**) 3.5 mm ultrasonically strengthened sample.

Comparing Figure 17a,b, it can be seen that after ultrasonic strengthening of the 2 mm-thick sample, the layers and the rasters have been fully fused, and the morphology of the rasters has been indistinguishable, indicating that the ultrasonic effect has penetrated through. For the entire sample, the temperature at the place where there is a forming defect inside the sample is heated up, and the adjacent rasters are quickly wound and re-fused, which makes up for the printing defect and improves the tensile strength significantly. Comparing Figure 17c,d, it can be seen that after ultrasonic strengthening of the 3.5 mm thick sample, some parts are well fused, but there are still defects of small bonding area of adjacent rasters and large pores. This indicates that the ultrasound effect is not sufficient, the ultrasonic transmission process inside the sample is greatly hindered, and the energy is weakened, so that some parts of the sample are not sufficiently fused, and the increase rate of the tensile strength is less than that of the 2 mm thick sample.

**Figure 18:**
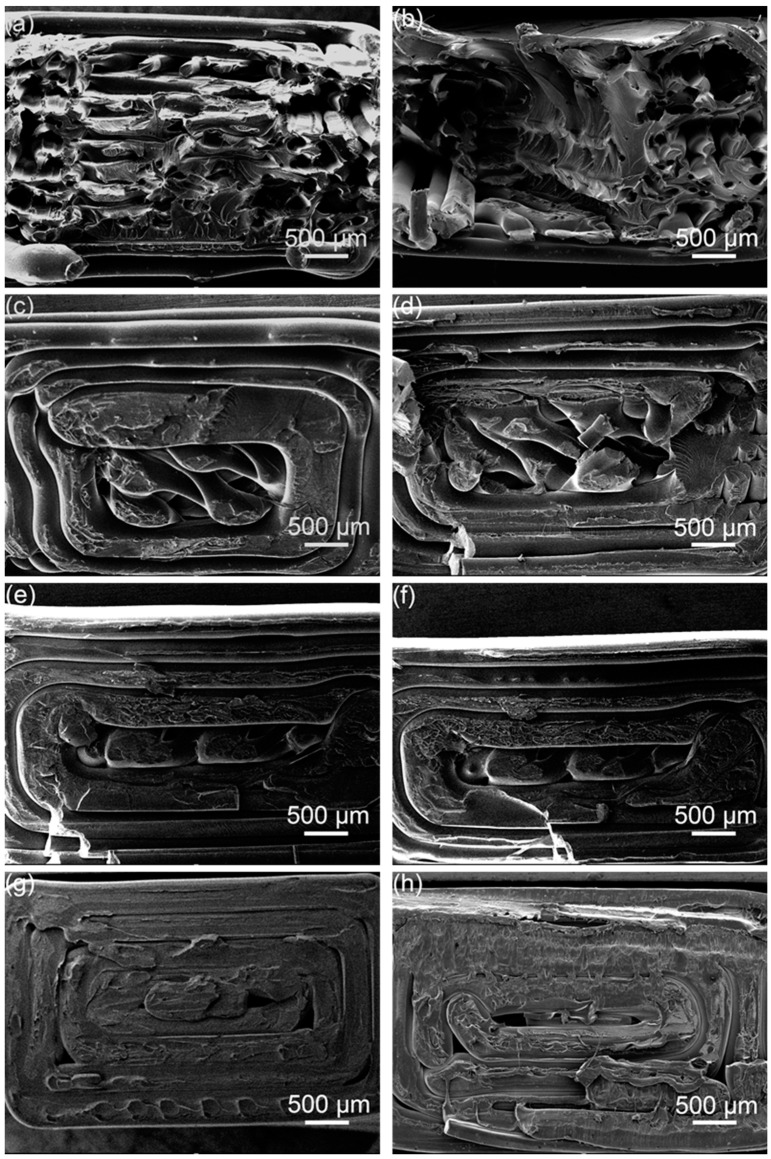
Electron micrograph of the original tensile cross-section at the printing angles of (**a**) 15°, (**c**) 45°, (**e**) 60°, (**g**) 90°; the printing angles are (**b**) 15°, (**d**) 45°, (**f**) 60°, (**h**) 90° tensile cross-sectional electron microscope images of ultrasonic reinforcements.

Figure 18 shows the tensile cross-sections of the samples at different printing angles before and after ultrasonic strengthening. Comparing Figure 18a,c,e,g, it can be seen that many broken filaments can be seen in the tensile section of the sample with a printing angle of 15°, and only the central part of the sample with a printing angle of 45° has broken raster material, and the tensile section of the samples with printing angles of 60° and 90° is flat, indicating that during the tensile test process, the strength of the raster material inside the sample with a printing angle of 15° greatly participates in resisting tensile deformation, and its tensile strength is relatively high. The sample with a printing angle of 45° may be due to the earlier slippage between the printed layers, which reduces the tensile strength; the samples with a printing angle of 60° and 90° are broken at the junction between the layers, which mainly rely on the adhesion of the raster to resist tensile deformation, and its tensile strength is relatively low. Comparing Figure 18a,b, it can be seen that after the sample with a printing angle of 15° is ultrasonically strengthened, multiple single rasters inside it are fused together to form a fusion zone, which increases the maximum load that the unit area can withstand, thereby the mechanical properties of the sample have been significantly improved. Comparing Figure 18c,d, it can be seen that after ultrasonic strengthening of the sample with a printing angle of 45°, the outline of the outer ring of the tensile section becomes rough, indicating that the ultrasonic increases the bonding area between the printed layers and the bonding strength increase, so the tensile strength has a certain increase. Comparing Figure 18e,f, it can be seen that the tensile section of the sample with a printing angle of 60° has no change after ultrasonic strengthening, and it is still relatively flat, indicating that ultrasonic has little effect on improving the interlayer bonding force. Comparing Figure 18g,h, it can be seen that the gap between the filaments on the printing layer becomes smaller after the sample with a printing angle of 90° is ultrasonically strengthened, indicating that the filaments on the same printing layer are fused, but the layer and the bonding strength between the layers are not significantly improved, while the tensile strength of the sample with a printing angle of 90° mainly depends on the interlayer bonding force between the printed layers, so the increase in tensile strength of samples with a printing angle of 90° by ultrasound is relatively limited.

### 3.2. Research on Additive Manufacturing Based on Ultrasonic Welding

#### 3.2.1. Mechanical Properties Study

(1) Ultrasonic welding of FFF samples

**Figure 19:**
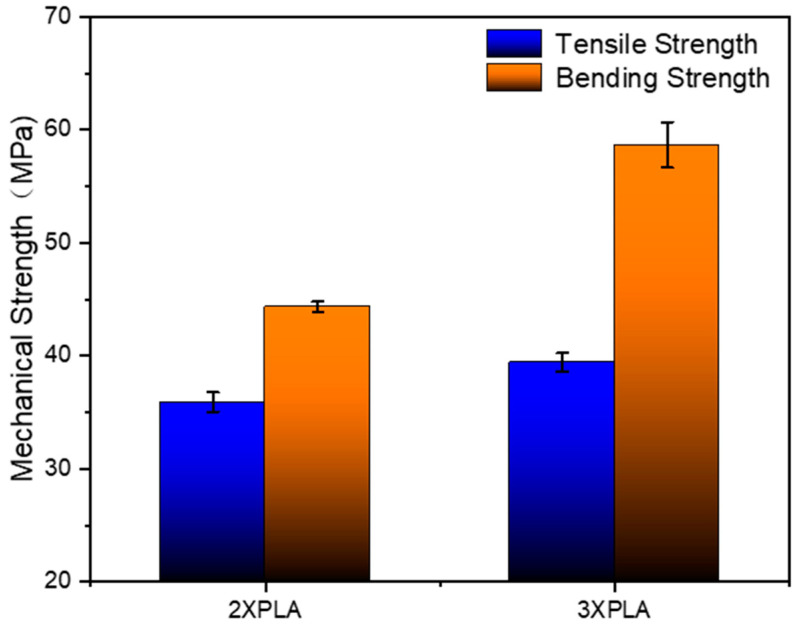
Mechanical strength of ultrasonic welded parts.

As shown in Figure 19, the tensile strength of the two-layer PLA weldment is 35.92 MPa, and the bending strength is 44.34 MPa; the tensile strength of the three-layer PLA weldment is 39.43 MPa, and the bending strength is 58.68 MPa. Compared with the two-layer PLA weldment, the tensile strength and flexural strength of the three-layer PLA weldment are significantly improved, which are increased by 9.77% and 32.34%, respectively, and the increase in the bending strength is higher than that of the tensile strength.

**Figure 20:**
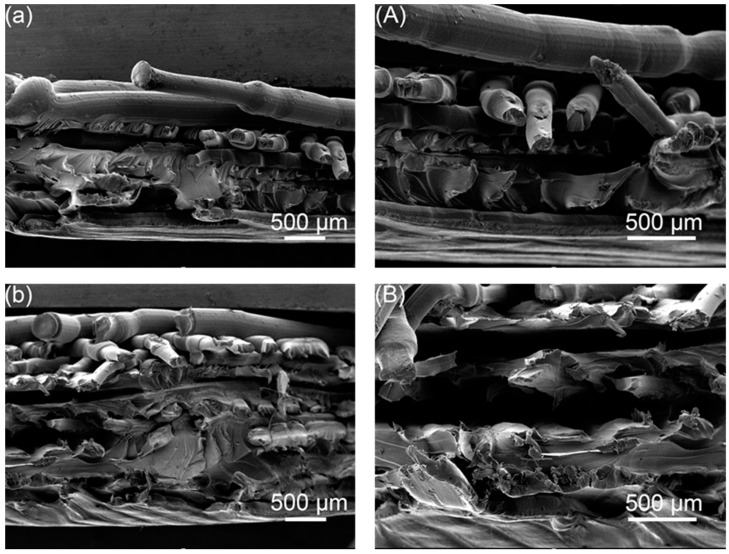
Tensile cross-sectional electron microscope image of the sample, 2-layer PLA ultrasonic welded part: figure (**a**) 30 times (**A**) 50 times; 3-layer PLA ultrasonic welded part: (**b**) 30 times (**B**) 50 times.

As shown in Figure 20, it can be found that the joints between the layers of the sample are well fused as a whole, and the contact surfaces of the sample have been fused into one, forming a strong molecular chain. In the process of layer-by-layer ultrasonic welding additive molding of the FFF samples, the samples are also strengthened layer by layer, which avoids the obstruction of ultrasonic waves due to the large thickness of the samples. It can be seen that the contact surface fusion area between the three-layer PLA welded parts is large, and the ability to resist tensile deformation is stronger.

Compared with PLA welding parts without metal embedded, the overall tensile strength is improved after metal copper is embedded, but the overall bending strength is decreased, because the metal copper hinders the contact between PLA, and the welding area is reduced, the delamination phenomenon is prone to occur during the bending deformation process. For the tensile strength of metal-embedded PLA weldments (Table 7), the samples with the same number of layers embedded in metal Cu1 are slightly lower than the samples embedded in metal Cu2, which may be due to the large pore size of Cu1, which is prone to fracture at the circular hole, resulting in slightly lower tensile strength; but for the bending strength of metal-embedded PLA weldments, the samples with the same number of layers embedded in metal Cu1 are higher than the samples embedded in metal Cu2. The connection strength formed by the hole is high, and the interlayer bonding force of the sample is also higher, so the bending strength is higher. In the process of metal-embedding polymer, the position and size of the aperture should be selected to ensure that the molded sample has relatively high mechanical properties.

#### 3.2.2. Shape Memory Performance Research

**Figure 21:**
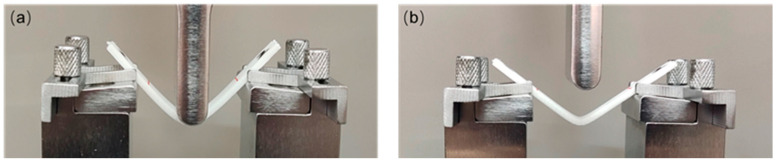
(**a**) External force is applied to the sample to a displacement of 15 mm. (**b**) 10 min later, the elastic deformation of the sample is restored after the external force is removed.

**Figure 22:**
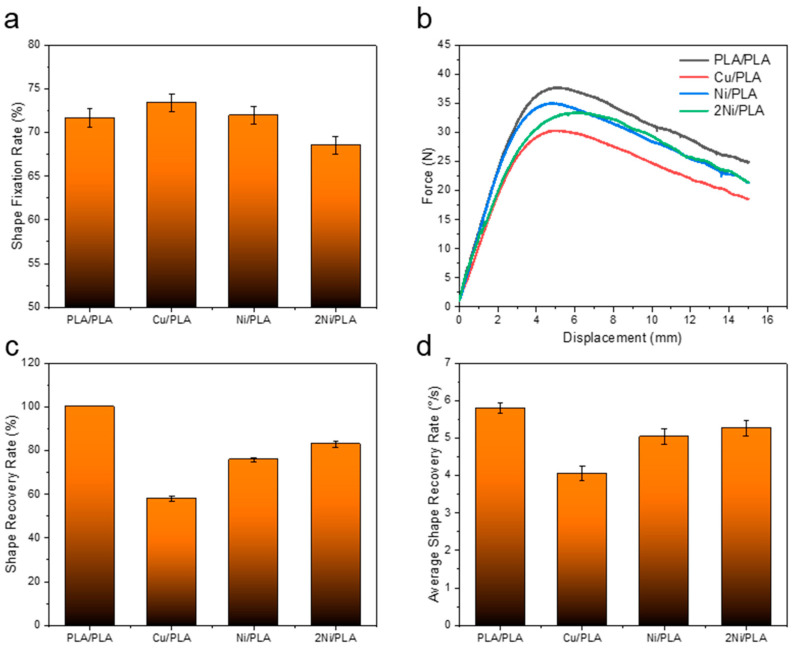
(**a**) The shape fixation rate of the sample. (**b**) The curve of force versus displacement during the bending deformation of the sample. (**c**) The shape recovery rate of the sample. (**d**) The average recovery rate of the sample shape.

As shown in Figure 21, at room temperature, the sample is deformed under the action of external force, when the external force is unloaded, the elastic deformation of the sample is recovered, and the sample is plastically deformed. As shown in Figure 22a, the shape fixation rates of the samples PLA/PLA, Cu/PLA, Ni/PLA, and 2Ni/PLA were 71.68%, 73.41%, 71.96%, and 68.55%, respectively. Among them, the shape fixation rate of the sample encapsulated with metal copper is higher, because the elasticity of copper is poor, which has a certain inhibitory effect on the elastic deformation recovery of PLA; the shape fixation rate of the sample encapsulated with two pieces of Ni-based amorphous alloy is low, because the elasticity of Ni-based amorphous alloy is better, which plays a key role in the recovery of PLA elastic deformation. As shown in Figure 22b, during the bending deformation process, the samples all reached their bending strength. It can be found that after the metal is encapsulated in the PLA, the maximum bending load of the sample becomes smaller. The reason for this is that the metal reduces the contact area of the upper and lower layers of PLA, reduces the welding strength, and weakens the ability to resist bending deformation.

**Figure 23:**
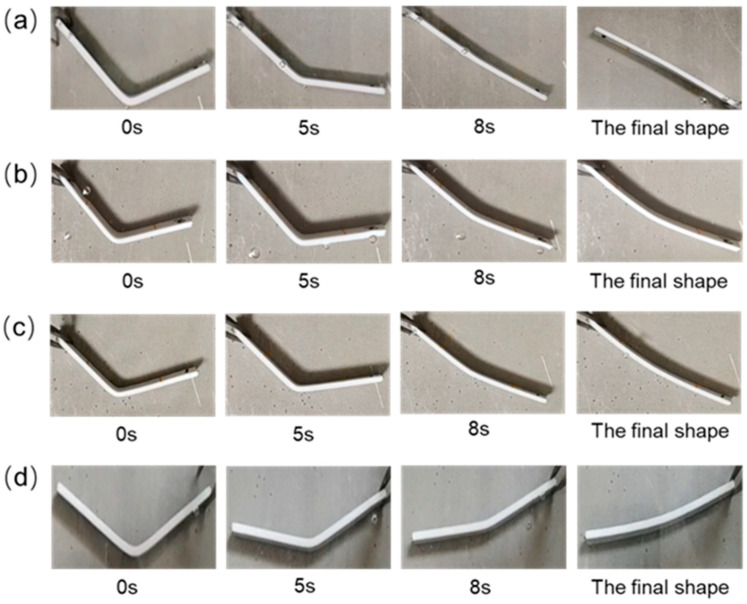
Recovery of the sample in a hot water bath, (**a**) PLA/PLA, (**b**) Cu/PLA, (**c**) Ni/PLA, (**d**) 2Ni/PLA.

It can be found from Figure 23 that the sample can be recovered quickly in a hot water bath at 70 °C. Figure 22c illustrates that the shape recovery rates of the samples PLA/PLA, Cu/PLA, Ni/PLA, and 2Ni/PLA are 100%, 58.06%, 75.86%, and 83%, respectively. It can be seen from Figure 22d that the average shape recovery rates of PLA/PLA, Cu/PLA, Ni/PLA, and 2Ni/PLA were 5.8°/s, 4.06°/s, 5.04°/s, and 5.27°/s, respectively. Among them, the PLA weldment can quickly and completely recover to the state before bending deformation. At room temperature, the PLA weldment is in a glass state, and the internal molecular chain basically does not have any movement. Under the action of external force, the molecular chain will be forced to move and deform, the deformed sample is placed in an environment above its glass transition temperature Tg, and the molecular chain will move to recover the deformation. Since the sample is placed in the water bath, the heat conduction is relatively fast, so the movement ability of the molecular chain in the PLA will be rapidly enhanced, and the deformation will be recovered quickly. In the process of PLA recovering and deforming, its restoring force will drive the metal encapsulated inside to recover together. The shape recovery rate and shape recovery rate of the samples encapsulated with Ni-based amorphous alloys are better than those of the samples encapsulated with copper. The reason is that the resilience of Ni-based amorphous alloys is significantly better than that of metal copper, and, driven by the restoring force of PLA, the ability to recover deformation will be stronger.

#### 3.2.3. Gear Sample Forming

**Figure 24:**
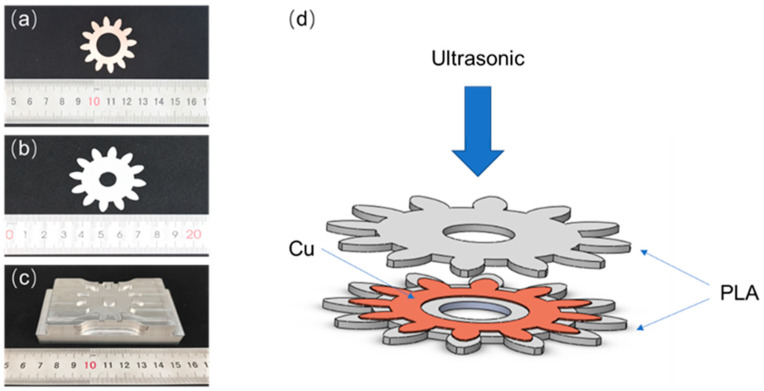
Ultrasonic welding gear prototype forming; (**a**) Metal gear, (**b**) PLA gear, (**c**) Ultrasonic plastic welding machine bottom mold. (**d**) Schematic diagram of gear forming.

Fused deposition molding gear samples of PLA material, customized bottom mold for gear welding as shown in Figure 24c, according to the above-mentioned method of using ultrasonic plastic welding principle to consolidate polymer sealing metal and accumulate additive manufacturing method, metal gears were encapsulated in PLA gears as shown in Figure 24d to form gears with metal inside and PLA outer shell. The gear parameters are shown in Table 8.

When the formed gear-shaped sample is deformed, it can be heated and recovered under the driving of the PLA shell. The metal copper inside has good electrical conductivity, which can be further researched and applied in the field of electronic components.

## 4. Conclusions

This paper combines FFF additive manufacturing technology with ultrasonic plastic welding technology. The strengthen mechanism of the samples’ mechanical properties are analyzed in depth. The bonding effect of ultrasonic on FFF two-dimensional samples is studied; the samples were strengthened and welded layer by layer, and the metal was sealed in the polymer. The Hybrid additive manufacturing of heterogeneous material parts combined with metal and polymer was realized, at the same time, its mechanical properties and shape memory properties were analyzed. Ultrasonic strengthening not only re-fused the pores inside the sample, but also improved the bond strength between the rasters. However, repeated application of ultrasonic waves will adversely affect the tensile strength of the samples. With the increase in the thickness of the sample, the increase rate of ultrasonic to the strength of the sample will decrease significantly. The effects of ultrasound on the interlayer adhesion of samples with various printing angles were different. The metal tape with round holes was embedded in the polymer PLA, and it was found that the size and density of the metal round holes had a certain influence on the mechanical properties of the molded sample. It was found that the recovery rate and recovery speed of the samples encapsulated with nickel-based amorphous alloys were better than those of the samples encapsulated with metal copper. The hybrid additive manufacturing method of ultrasonic and fused filament deposition provides a new approach to high-performance additive manufacturing of heterogeneous materials.

## Figures and Tables

**Table 1:** Research on strengthening mechanical properties of FFF samples.

Strengthening Mechanical Properties of FFF Samples	Methods
Carbon fiber reinforced composites were prepared by adding different content of carbon fiber into ABS	Composite reinforcement
ABS/OMMT (organic modified montmorillonite) nanocomposites were prepared	Composite reinforcement
The effects of construction direction, layer thickness and feed rate on mechanical properties of fused deposited PLA were studied	Optimization of print parameters
The influence of the forming direction and forming angle on the mechanical properties of FFF samples was studied	Optimization of print parameters
ABS-RS (straw) composites were developed for FDM process	Composite reinforcement

**Table 2:** Experimental design.

Experiment Number	Variable	Numerical Value	Unit
1	Ultrasound enhancement times	0, 1, 2, 3, 4	Frequency
2	Sample thickness	2, 2.5, 3, 3.5	mm
3	Print angle	15, 30, 45, 60, 90	°

**Table 3:** Printing parameters.

Parameter	Numerical Value
Printing speed	60 mm/s
Extrusion speed	45 mm/s
Nozzle temperature	195 °C
Print layer thickness	0.2 mm
Cover solid layer	4
Outer ring solid layer	3
Fill line spacing	0 mm

**Table 4:** Ultrasonic process parameters.

Parameter	Numerical Value
Vibration frequency	20 KHz
Welding pressure	3.5 kg/cm^2^
Welding time	0.35 s
Delay time	0.49 s
Curing time	0.5 s

**Table 5:** Sample material and size.

Sample Material	Sample Size
PLA	1 mm × 75 mm × 10 mm
Metal copper	0.04 mm × 75 mm × 3 mm
Ni-based amorphous alloys	0.04 mm × 75 mm × 3 mm

**Table 6:** Sample thickness reduction rate statistics.

Sample	Thickness Reduction Rate
Once ultrasonic sample	9.88%
Twice ultrasonic sample	14.38%
Three-times ultrasonic sample	19.09%
Four-times ultrasonic sample	20.43%

**Table 7:** Tensile strength and flexural strength of metal-embedded PLA weldments.

Sample	Tensile Strength (MPa)	Bending Strength (MPa)
3-layer Cu1	37.83	48.62
3-layer Cu2	39.78	46
5-layer Cu1	42.24	40.36
5-layer Cu2	43.01	31.07

**Table 8:** Gear Parameters.

Gear Parameters	PLA Gear	Metal Gear
Modulus	3	2.5
Number of teeth	12	12
Pressure angle	20	20
Face width	1	1
Nominal shaft diameter	10	15

## Data Availability

The data presented in this study are contained within the article.
